# Rapid eigenpatch utility classifier for image denoising

**DOI:** 10.1038/s41598-025-96859-x

**Published:** 2025-05-13

**Authors:** Michael A. J. Mitchell, Stefano Sanvito, Lewys Jones

**Affiliations:** 1 Centre for Research on Adaptive Nanostructures and Nanodevices, Dublin, D02 W085 Ireland; 2 Advanced Microscopy Laboratory, Dublin, D02 DA31 Ireland; 3 School of Physics, Trinity College Dublin, Dublin, Ireland

**Keywords:** Materials science, Nanoscale materials, Techniques and instrumentation, Computational science, Software

## Abstract

Under low-illumination conditions, images inevitably contain both Poisson and Gaussian noise. In electron microscopy, there is the added complication whereby increasing the dose-rate, to improve signal-to-noise, damages the specimen being imaged, making certain materials being impossible to characterise. Conventional data smoothing techniques may dampen usable image contrast, and deep-neural network (DNN) based approaches risk the introduction of artefacts. In this work, the complementary strengths of patch-based and DNN approaches are combined into a lightweight denoising architecture such that experimental data integrity is preserved while effectively removing noise. Our approach, the Rapid Eigenpatch Utility Classifier for Image Denoising (REUCID), leverages the speed and data-integrity of a non-local patch-based SVD step to identify key image components, followed by a convolutional neural network (CNN) acting strictly in a classification capacity on the SVD eigenvectors. This classification-only approach to DNN integration represents a significant advance by mitigating the risk of DNN overreach while maintaining denoising effectiveness. We demonstrate superior performance on high angle annular dark field images, where our hybrid method outperforms conventional techniques in enhancing image contrast while preserving genuine structural features.

## Introduction

When imaging beam-sensitive materials, it is usually advisable to use the minimum beam intensity possible^1^, this is often referred to as so-called “low-dose imaging”. However, using low dosages creates a number of unavoidable problems; from hindering the operators navigation/alignment/focusing to obfuscating finer image details. In most microscopes there will be Gaussian-like thermal noise, and even if this can be overcome, then eventually Poisson (shot) noise will dominate to such an extent that characterising the sample may become impossible. Where beam-sensitive specimens are imaged, the intensity used should yield the bare minimum of signal to obtain a computer-assisted measurement with the necessary reliability; but then this “ideal image” will always appear noisy to human perception.

In this work, we focus on a Poisson limited process with Gaussian noise in a high-resolution Scanning Transmission Electron Microscope (STEM) is where the dose problem is most acute^2^, though of course the low-dose problem persists in other imaging regimes too^3,4^. As readout technology tends towards the Poisson limit, both in the context of electron microscopy^5^ and optical sensors more broadly, any in-field denoising approach must be robust to shot noise.

The unavoidable problems associated with low-dose imaging necessitate a fast denoising algorithm that can assist the operator at the microscope. Current fast denoising solutions rely on one or more intrinsic assumptions or priors, and hence risk some deleterious property. These might be the introduction of artefacts in real-space from Fourier-domain processing^6^, or a loss of temporal-resolution from rolling frame-averaging.

For images that contain either repeating areas of similar feature/texture, or for images whose ground-truth has some smoothly varying form, non-local image denoising has shown impressive performance^7^. Using this approach in conjunction with sparse-based methods can prove very effective, and further relying on sparse representations over dictionaries (learned or analytical) can improve speed^8^. This must be considered in the context of the invigoration of the field of denoising in light of deep neural networks (DNNs)^9,10^. The DNN denoising paradigms includes^11^ generative models (those which rely on generative adversarial networks^12,13^ or variational autoencoders^14^ for example), and convolutional neural network (CNN) based methods^15^. This is an area of very active development, with additional works demonstrating the performance of transformer architectures for image denoising, most notably the Restormer architecture^16^. Another DNN approach that demonstrates a sensitivity to the risk of artefacts and hallucination is a recent work by Li et al., which incorporates the fundamental physics of light as an inductive bias to inform the recovery of data^17^. A different approach which also aims to mitigate artefact introduction through the use of an invertible mapping to a latent space wherein an unsupervised transformation occurs is the recent work by Kwon et al.^18^. The most relevant (applied to electron microscopy), highly performing, generative-denoiser which we compare to as a state-of-the-art is that of a recent Lobato et al.^19^. In spite of advancement of DNNs and the generative approach to denoising, the fact remains that patches from well-sampled images over-determine image features, lending the problem well to dimensional reduction^20,21^, especially for somewhat self-similar images^22,23^. We look to combine the advantages of classical patch-based denoising, with the flexibility afforded by the DNN paradigm in such a way that the experimental data is respected and remains free from artefacts.

We layer on the older patch-based approaches using a semi-supervised machine learning (ML) framework. Our architecture structure leverages the generalisation abilities of ML to tackle the ambiguities in the unsupervised (Singular Value Decomposition (SVD)) output, while keeping the role of the ML component narrow enough to prevent it over-imposing on the raw data, leaving the microscope operator a faithful representation of the experimental data at hand. The efficacy of layering on an unsupervised (Principal Component Analysis) PCA/SVD step with a further supervised filtering step has been well demonstrated in a noise profile identification context^24^. There has also been work investigating the SVD/CNN combination in a noise removal context^25^, whereby the architecture first uses a convolution to extract features, followed by a PCA step. However, this approach relies on further convolutions in the reconstruction. , To minimise the risk of introducing artefacts and non-physical distortion towards, while still aiding the technician at the microscope, we avoid the use of DNN techniques in the reconstruction.

The main contributions of this work can be summarised as follows: (1) We introduce REUCID, a novel hybrid architecture that combines the advantages of traditional patch-based methods with the discriminative power of neural networks without compromising data integrity; (2) We demonstrate a new approach to neural network integration in denoising workflows where the deep learning component is strictly limited to a classification role, preventing the introduction of non-physical artefacts; (3) We implement an adaptive patch-size determination method that standardizes feature scale presentation to the deep learning component (4) We provide experimental validation on both simulated and real microscopy data across a range of dose conditions, demonstrating the ability to preserve genuine structural features while effectively removing noise. (5) We outline potential avenues for further development.

## Methods

### Overview

**Fig. 1:**
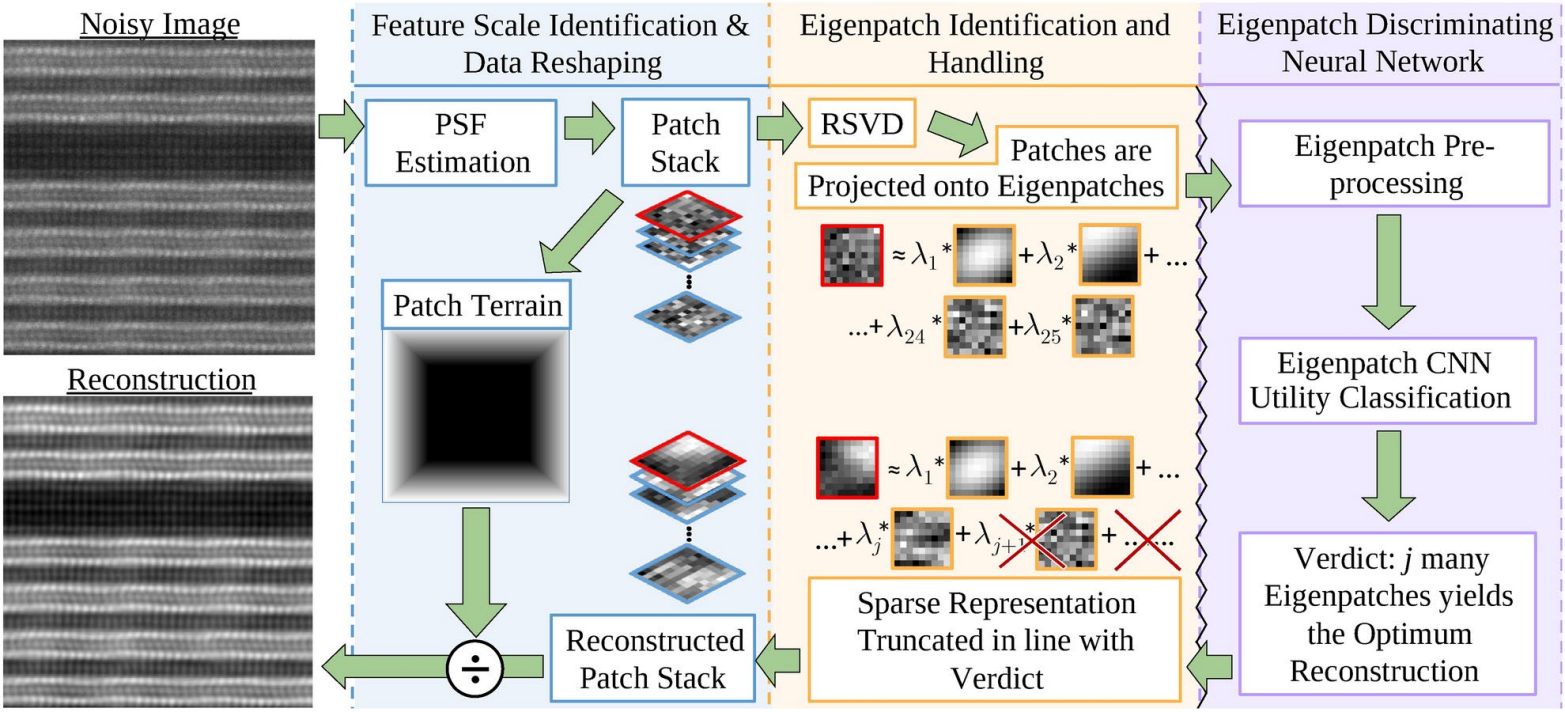
The REUCID architecture, described in detail in the main text. It is important to note that the image data never passes the jagged black line in or out of the deep-learning section, ensuring data-integrity.

Here, we introduce the dataflow of the algorithm, which is altogether summarised in Fig. 1. The motivations will be discussed in the following paragraphs. First, the size of the Point-Spread-Function, which is the presentation of point-like, sub-resolution objects in from an optical system in the images it produces, is dynamically determined; this will be the size of the image patches. The patches are extracted from the image in separate patch-stacks rather than all at once, where each stack is sufficiently small such that it may be processed entirely within the available RAM. In the processing of a given stack, an SVD step is performed. Each patch in the stack is expressed as a linear sum of the SVD eigenvectors. The “utility” (described in the following paragraph) of the SVD eigenvectors are classified by a CNN. The aforementioned linear sum is truncated in line with the CNN classification verdict. The reconstructed patch stack is placed back onto the image canvas. This is repeated for each stack. The patch overlaps are calculated, and the image is divided out by this overlap matrix, delivering the reconstructed image. The pseudocode for this process is given in Algorithm 1.

**Algorithm 1:**
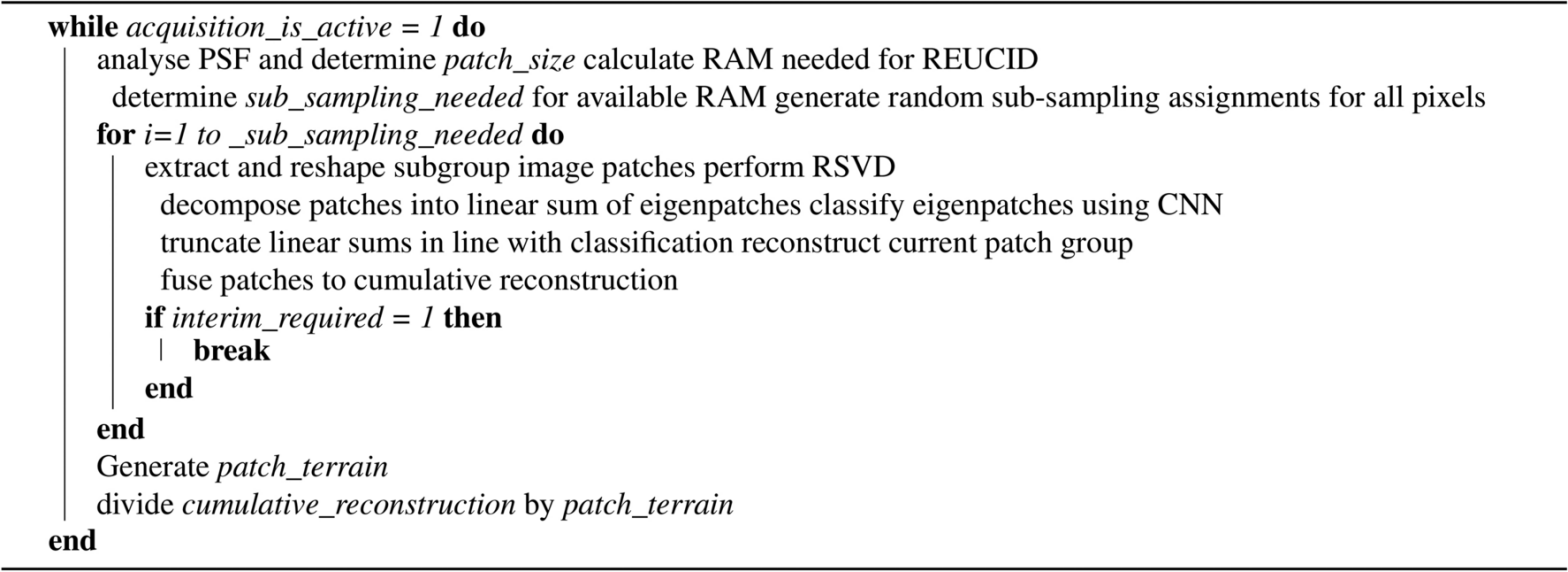
REUCID.

**Fig. 2:**
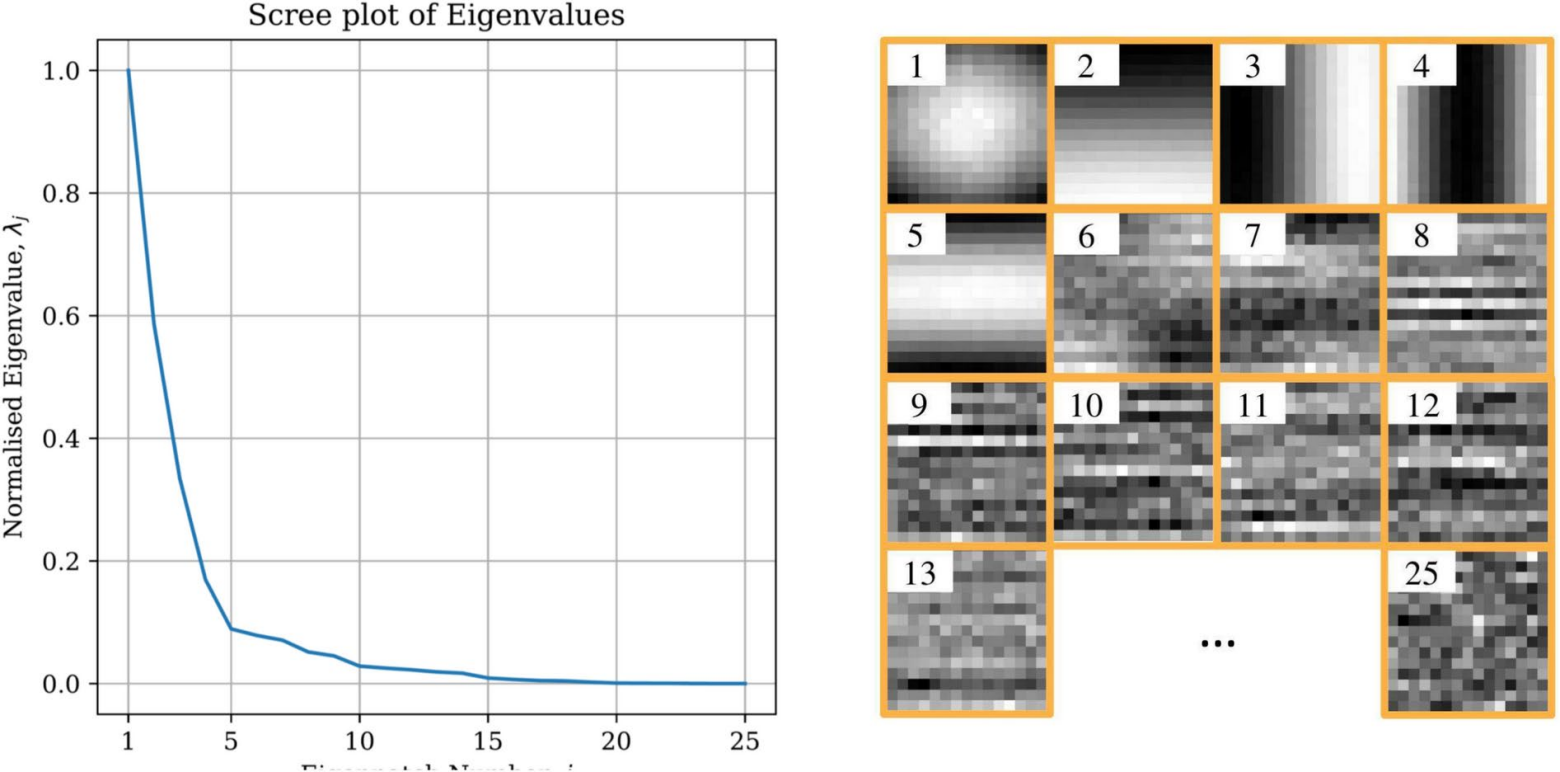
A figure demonstrating the decreasing relevance of the RSVD-determined eigenpatches in capturing genuine image detail, as reflected in the decrease in associated eigenvalues.

The SVD eigenvectors, referred to from here on as “eigenpatches”, represent the patch components that are universal to all patches in the stack. The greater the magnitude of the SVD eigenvalue corresponding to a given eigenpatch, the greater the importance of that eigenpatch in describing the patches in the stack. For low-rank data such as electron microscopy images, there comes a point where most of the image features have been captured by higher-importance eigenpatches, leaving only the image features that are highly variable and specific to individual patches, i.e. noise, to be discerned by the remaining lower importance eigenpatches. By discarding these eigenpatches in the reconstruction, the image will be denoised. There is no literature consensus on a general approach to identifying these low-importance eigenpatches that detract from useful image contrast in the reconstruction. The scree plot of eigenvalues is ambiguous as to where the cutoff should be, Fig. 2. One might try feature engineering on the eigenpatches in an attempt to facilitate clustering between useful and useless eigenpatches. We tried unsuccessfully to use heuristics such as the gradient and entropy of the eigenpatches represent the data to induce such a clustering, though these constructions did not generalise well to clustering satisfactorily on different materials. It is at this point where the ML component is employed. We use a CNN as a classifier of the eigenpatch utility, where the CNN is functioning effectively as a flexible clustering mechanism since the conventional heuristics have failed.

This SVD/CNN combination approach has a number of advantages; firstly, the data is represented in a manner more native to the image patterns at hand^26^ due to the SVD step, rather than forcing a dictionary representation on the data, effectively optimising the sparse method by leveraging self-similarity as in^27^. Most importantly however, the CNN component only interacts with the data as a classifier on the SVD eigenvectors, reducing the issue of artefacts being introduced from the training set as is risked where the ML component interfaces directly with the denoised output in a generative capacity. By taking this approach, we retain data integrity without losing speed. Also, it has long been acknowledged that the best sparse-based methods which rely on dictionaries often require tailored dictionaries^28^. Here, the training of the ML component tailors it to the general point spread function patterns within the electron microscopy imaging domain, which when coupled with the SVD step’s adaptability in representing the data, enables the dataflow to function as a flexible analog to the dictionary approach.

One of the main issues with patch-based approaches is the risk of over-smoothing^29^. This is reason for the emphasis on finding the right cutoff; if the patch representation in terms of stack eigenpatches is truncated too early, finer image detail will be lost, which will present as over-smoothing in the reconstruction. There is also the possible burden of execution time, which is why the algorithm was built to function within RAM, together with the ability to return interim results without having to process every patch stack. A real-time performance analysis is not performed in this work.

Though acknowledging the utility of image priors with respect to computational speed^30^, in the experimental context it is preferable to edit the data as little as possible, and so the use of a preparatory data smoothing, through techniques like total variation regularisation or imposing a low/maximum entropy, is avoided. This is also important for trust within the imaging community for an algorithm to be adopted. This is not to say that a priori knowledge is not used; this architecture does leverage image self-similarity to derive the optimum patch size (though not necessarily requiring the strongest form of image self-similarity, periodicity, as used in^31^). However, this a priori assertion is not used to impose on the data in a certain manner, but only to present the data to the denoising component of the algorithm in appropriately sized chunks. The relevance of this adaptive capacity will be explained in a later section.

The proposed algorithm does not attempt to correct for sample dynamics presenting through blur kernels^32^, though denoises the image such that subsequent image corrections might be more effective. For example, there is an additional factor to consider when denoising serial-scanned data such as Annular Dark Field (ADF)-STEM; scan-noise^33^. The experimentalist must decide whether to treat high-frequency scan-noise as either genuine data or as an artefact to be removed. This decision depends on the purpose of the study. For example, when observing high-frequency atomistic dynamics, retaining this effect may be desirable. However, in laboratories well-shielded from deleterious acoustics or in ultra-low-dose imaging where shot-noise dominates, removing this noise better reveals the sample’s static features^34^.

### PSF estimation

All optical systems have an ultimate image resolution that is determined by several factors, including the radiation wavelength, aperture choice, lens aberrations, finite source size, pixel-density (spatial sampling)^35^, and so on. Conveniently, once the image-data is recorded, all of these parameters act collectively and behave as a single finite Point-Spread-Function (PSF). Radiating sources that are sub-resolution are functionally point-like to the optical system, and the PSF is the associated spatially-extended blob that represents these point-like objects in the resulting images.

The REUCID algorithm was derived in the context of High Angle Annular Dark Field (HAADF) electron microscopy, wherein roughly similar PSFs, are translated about the image. Crucial to the denoising performance of REUCID, for reasons explained later, is the patch-size used; roughly the diameter of the PSF. Modelling of the PSF itself is excessive, both in its exactness in the context of the determination of an ideal patch-size in pixels, and in light of the goal of minimising computational cost. This built-in adaptive capacity is crucial; patches that are too large with respect to the image’s self-similary unit, the PSF, will lead to excessive RAM and CPU use, while patches which are too small, will lead to the identification of too few unique components in the SVD step, leading to poorer noise removal or poorer retention of genuine image detail^36^.

The autocorrelation function (ACF) can be used as a fast and sufficiently precise measure of the PSF width (not necessarily PSF shape). The intuition behind the ACF is relevant here; one copy of the image is displaced relative to a second copy, and the degree of similarity of just the overlapping parts dictates the value of the autocorrelation at that point. The first minimum of the radial average of the ACF corresponds to the distance at which the PSFs of the first copy completely fail to overlap with the PSFs of the second. The first inflexion is used if the radial average fails to admit a minimum due to an aperiodic image. The circular convolution theorem is used to speed up the computation via Fast Fourier Transforms, since this transforms the convolution to a computationally less expensive point-wise product^37^.

### RAM estimation

The algorithm is structured so that calculations may be handled within the RAM of a standard desktop. For an image of size $m \times n$ pixels, divided into square patches of PSF-width for odd integer *p*, a total of $(m- p+1)\times (n- p+1)$ patches will be generated. This formula works by multiplying the number of valid horizontal starting positions $(n- p + 1)$ by the number of valid vertical starting positions $(m- p + 1)$ to get the total number of possible patch positions in the image, where the “+1” is needed because you can start a patch at both the beginning and end positions along a row/column. This formula is under the assumption of no image padding, which would introduce distortions into the analysis. Each of these patches will have area $p^2$ pixels. Where we are interested in processing large images, and where *p* is much smaller than *m* or *n*, the total amount of patch data we expect will scale with approximately $(m- p+1)\times (n- p+1)\approx m\times n\times p^2$. This expression is also the size of the array produced when the square patches are reshaped to vectors ready for the SVD.

If we consider a typical survey image of say $512 \times 512$ pixels, with a patch size of 11pix, then this becomes 31.7 million elements. For double-precision numbers with 64bit depth each, this would require 254Mb RAM just to hold the reshaped array. Having an array which would hold the decomposed results can be circumvented, reducing memory requirements further, in a process described later. In practice still more RAM would be needed during the SVD step.

If the STEM operator were to then record a frame at (typically) $1024 \times 1024$ pixels, the equivalent patch would be $\approx 23$pix, and the total RAM needed would be 4.5Gb and beyond older PCs stationed at many electron microscopes. Moreover, the data-transfer overhead when reading/writing to this RAM would likely make this computation impossible to run live. To remedy this problem, the expected RAM use is calculated in advance using the known image and patch dimensions. By comparing this with the RAM available to the image-processing software environment (necessarily less than the whole system RAM), we can determine the number of workload division, *d*, needed.

### Patchisation

Integers from 1 to *d* are randomly assigned to every element in an array the size of the image. Patches are assigned to one of *d* patch stacks depending on the value of the integer element at the index of its top left-pixel. A loop is created to extract and process the suitably sized patch stacks in turn. Pre-clustering of similar image patches, as in^38^, is avoided to reduce computational cost. The last patch-size$-1$ rows and columns do not have patches drawn from them, since extracting their corresponding patches from the image would require padding past the edges of the image, which introduces artefacts into the reconstruction. However, despite not having a patch of their own, pixels within these rows and columns are still treated by the algorithm due to being included in the processing of other patches. This facilitates their denoising, which is important so that they need not be discarded in the reconstruction. This is in direct contrast to other approaches wherein the border cannot be denoised and so cannot be included in the reconstruction, losing a significant amount of data^34^, and an unacceptable loss of field of view. The is key because in experimental acquisition, the datum of each pixel represents a very real cost to the experimentalist.

Using the ensemble allows for overdetermination of the optical system’s PSF, and this repeated sampling of the same translated image feature allows for denoising of individual patches. The “patchisation” step is crucial to the algorithm’s functioning. Standardising the feature scale to be evaluated during classification by the neural network, by determining the patch-size via the PSF analysis, is what renders this adaptive patchisation approach immediately generalisable to different materials and PSFs. This is because the training set is very easily expanded since it does not need an understanding for global image structure to remain effective. There is also the benefit of patchisation discretising the workload so that the denoising algorithm may return interim results if speed is paramount.

### Unsupervised learning and pre-processing

The 3-dimensional patch-stack for a given workload division has its patches re-shaped as vectors that all together constitute a 2D matrix suitable for Singular Value Decomposition (SVD), specifically Randomised SVD^39^, a faster version. The flattened patch-stack vectors are projected onto the RSVD-determined eigenpatches in a sparse-coding step; expressing each patch approximately as a linear sum of eigenpatches, or in other words; individual patches are expressed as a unique combination of the shared traits among all patches. The eigenpatches are prepared for classification by first interpolating to a fixed size of 25px $\times$ 25px, required because of the fixed dimensionality of the ML component input. The SVD eigenvalue, a good but not sufficiently expressive predictor on its own of the eigenpatch utility, see Fig. 2, is passed alongside the interpolated eigenpatch for classification.

### Eigenpatch classification

A certain number of eigenpatches is absolutely necessary for faithful reconstructions of the patches, other eigenpatches capture no image detail, and contribute almost entirely noise. The difficulty arises with the ambiguous eigenpatches, those which perhaps have some utility, but also contribute considerable noise. Ending the sparse representation too early will fail to preserve the image fine scale texture structures^40^; ending it too late will retain noise in the reconstruction. The components are classified through a Convolutional Neural Network (CNN) as either being worthwhile for including in the reconstruction, or deleterious to image quality. The linear sums corresponding to the projected patch-stacks are truncated in accordance with the results of the CNN classification, leaving an expression for each patch in terms of the useful eigenpatches only.

### Dataset creation

Deciding the optimal number of eigenpatches to use in the reconstruction is not a truly objective process, since what is deemed an optimal reconstruction by the viewer may not coincide with the optimal reconstruction as indicated by the image denoising metrics (discussed later). To address this theoretical blindspot, we opted to deploy a survey to inform the ground truth of our classification approach. To generate the survey data, we considered a set of simulated and experimental electron microscopy images. For the simulated data, we used the Prismatic software package to generate high-resolution STEM images of Si[110], BaNdTiO, Graphene, and SrTiO3, at various electron dose rates (100, 500, 1000, and 10,000, and e/Å$^{2}$), together with Gaussian noise to simulate detector noise. For each of the afore-mentioned materials, we also simulated the “infinite”-dose, perfect image for reference. Each one of the noisy images in the set were then processed through the PSF estimation, RAM estimation, patchisation, and RSVD workflows, yielding a number of eigenpatch-eigenvalue pairs in turn. The survey involved 89 electron microscopy experts and 135 non-experts from various institutions. During the survey, for a given noisy image, the respondent was asked to choose the best reconstruction from a number of options. The options were constructed by using different numbers of eigenpatches to reconstruct the image after the RSVD step. The mean of their aggregated responses (using each response individually would introduce a large amount of redundancy in the dataset) for a given reconstruction was chosen to inform the optimal number of eigenpatches to be used, and hence the utility of each eigenpatch. It was found that the expert/non-expert distributions aligned very closely, Fig. 4, so both were used to inform the model.

The eigenpatch size would depend on the size of the PSF determined in the processing of a given image, and so in order to standardise the input to the CNN, the eigenpatches were interpolated to a 25px $\times$ 25px input. The eigenvalues were normalised relative to the maximum eigenvalue of their respective decompositions. This dataset of eigenpatches and their respective utility was augmented to enhance rotational invariance through rotations and inversions, effectively increasing the training data by a factor of 8. In total there are 4200 eigenpatch-eigenvalue-utility datapoints created, with an 80/20 train/test split used for training.

### Hyperparameter tuning

**Fig. 3:**
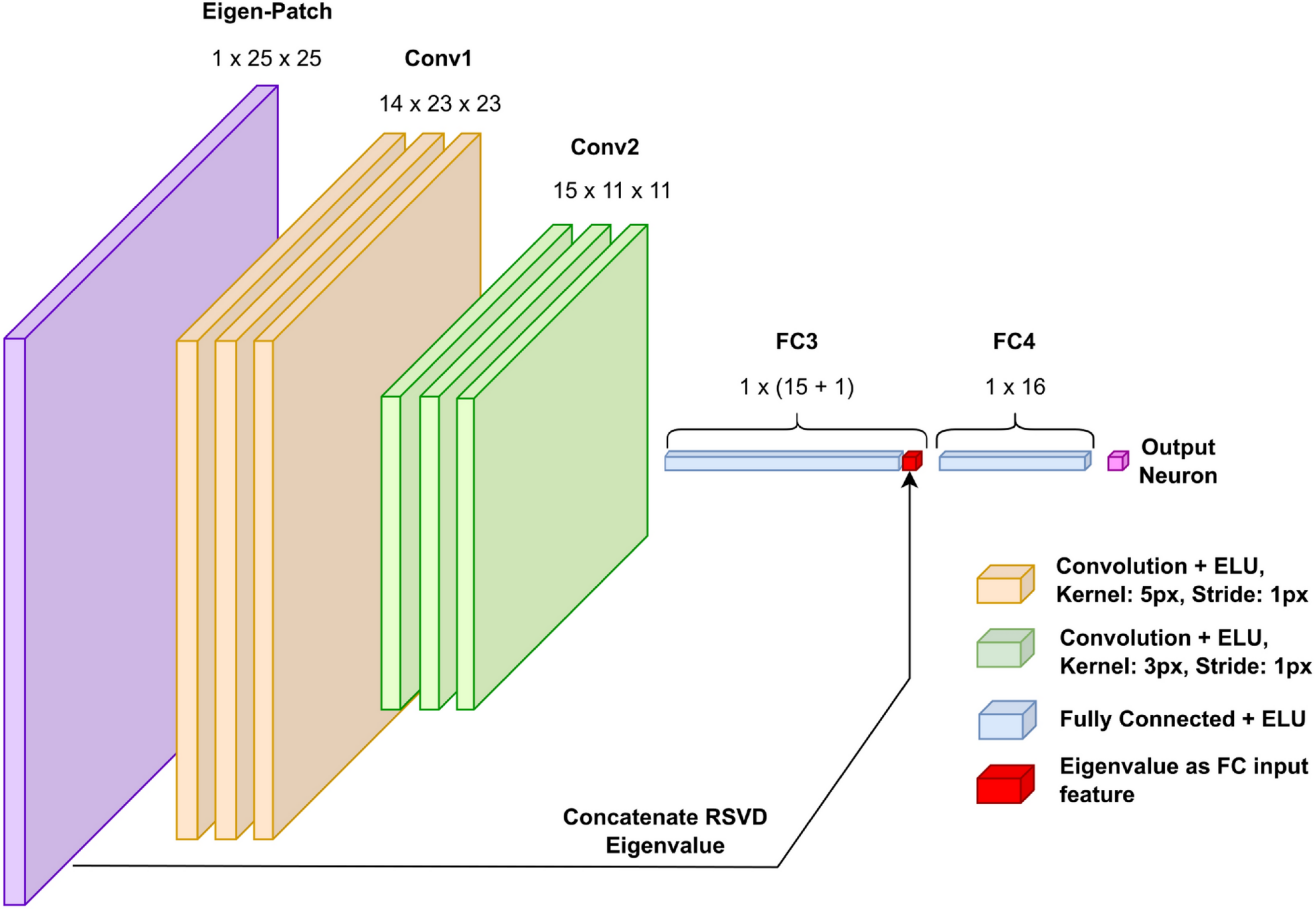
The ML component schematic. Conv1: A 14 channel, $5 \times 5$ kernel, 1-stride, 1-padding convolution is applied to the single channel input. This is followed by an ELU activation, and a $2 \times 2$, stride 2 max pooling step. Conv2: A 15 channel, $3 \times 3$ kernel, 1-stride, 1-padding convolutional layer. This is followed by an ELU activation, and another max pooling step. An average pooling is performed over each channel, and the RSVD-determined eigenvalue is appended to this vector of channel averages. This passes through width-16 ELU-activation dense layers, FC3 and FC4, before passing through a Sigmoid activation for binary classification of eigenpatch utility.

A very simple CNN model is used for the classification as a proof of concept, with the architecture structure laid out in Fig. 3, though a more elaborate ML architecture could well be more appropriate here. The convolution kernel size (5px $\times$ 5px) is quite large relative to the 25px $\times$ 25px input. This is to prevent against artefacts introduced from the interpolation to the fixed-size input to the CNN. If some image is best processed with a PSF-estimation determined patch-size of, say, 7 pixels, it is the case that for the noisy, poor-reconstruction-quality inducing, low-eigenvalue eigenpatches, that a “hot” pixel (a Poisson noise artefact not indicative of an image feature) would be interpolated in pre-processing to a high intensity blob roughly 3.5 pixels in diameter. The design idea here is that using a large CNN kernel for the first layer gives the kernel a greater ability to contextualise these unphysical blobs as not worth informing its determination of eigenpatch utility. The eigenvalue is added to the flattened output of the second convolutional layer before being sent through the 2 remaining fully connected layers. The number of channels and size of the linear layers was determined with a Bayesian optimisation using Optuna^41^.

### Patch terrain calculation

Densely sampling the image to “jointly filter image [patches]”^26^ means that parts of the image canvas which will have more patches placed onto it that others, a redundancy which must be accounted for and addressed^42^; we call this these overlaps the “patch-terrain”. It will be especially uneven in the context of our proposed architecture if the algorithm must exit before all patch-stacks are processed. Placing patch-sized arrays of ones onto the image canvas is not the most efficient way to calculate the terrain. Instead, a “rolling” calculation technique is used.

The rolling technique consists of binarising the array of random integers that determine which patches belong to which workload divisions, where any pixel for which the corresponding patch has been processed is one, and is otherwise zero. The height of the patch terrain at any given pixel depends on the number of top-left pixels that have been processed in the patch-sized region above and to the left of the pixel in question. We will call this region the trigger-region (TR), and the sum of over this region in the binarised array is what gives the patch-overlap.

With this in mind, the binary array is then bordered with zeros, a border of width patch-size. This is to handle pixels near the edges. Now we note that the sum calculated for the TR of given pixel shares much of its TR with the pixel to the right of it; the difference being one more patch-size column to the right of the original TR, and one less patch-size column being the original TR’s leftmost column. Creating a rolling sum where the aforementioned left column is subtracted and right column added when moving between adjacent pixels, this cuts down on the patch-size$^2$ many summations per patch-terrain pixel that are other-wise required towards linear scaling. This has been successfully implemented to work where the rolling sum moves from left to right, but could be further improved in future work to perform simultaneous vertical rolling sums also. The image is divided out by the patch-terrain, yielding the fully reconstructed image (Fig. 3).

### Software platforms & hardware specifications

**Fig. 4:**
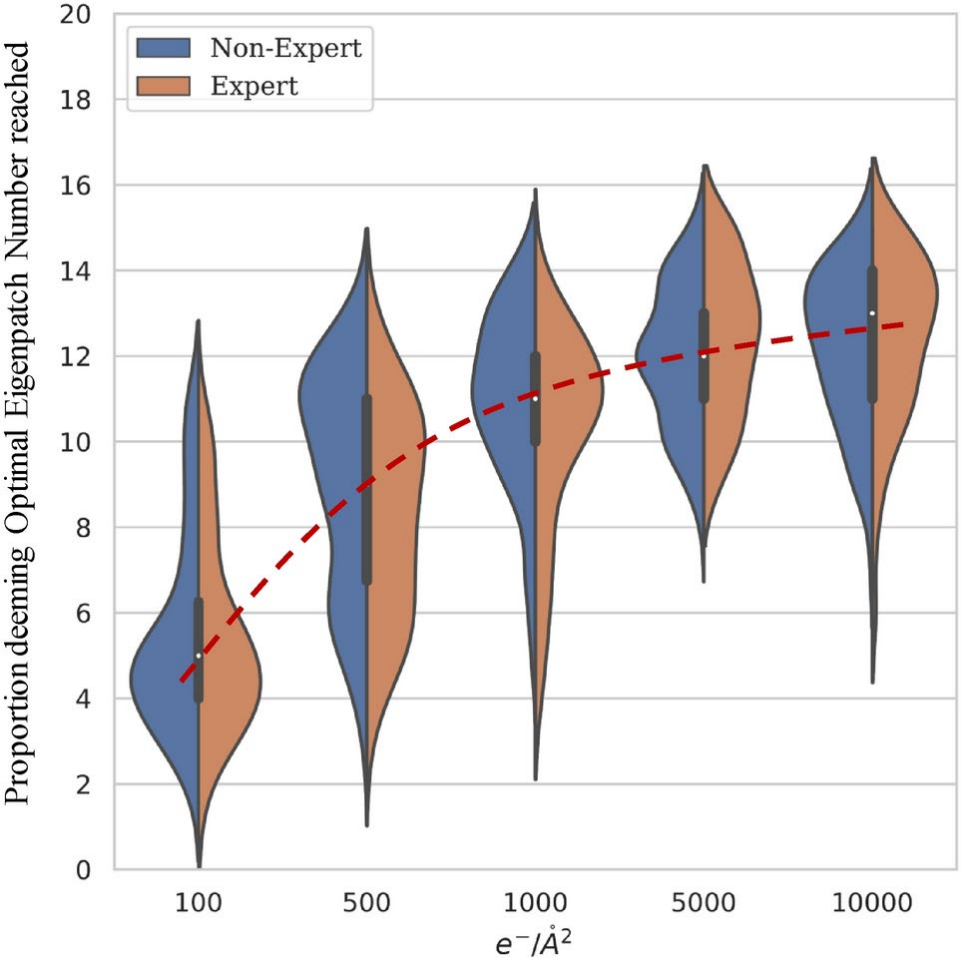
A violin plot of the expert vs. non-expert survey distributions. Note the gradual tapering off of the average response as dose increases.

The PSF determination, patchisation, RSVD steps, and patch terrain determination are written in Python using the Numpy library^43^. The CNN step is written in Pytorch^44^. The algorithm is not yet parallelised. The algorithm does not make use of a GPU for any stage of the algorithm, though there are components which would experience a significant speed-up from such a rewrite. The interim calculations are performed using 64 bit array values, so in an operational version the numerical precision would be reduced (Fig. 4).

## Results

### Restorative performance

**Fig. 5:**
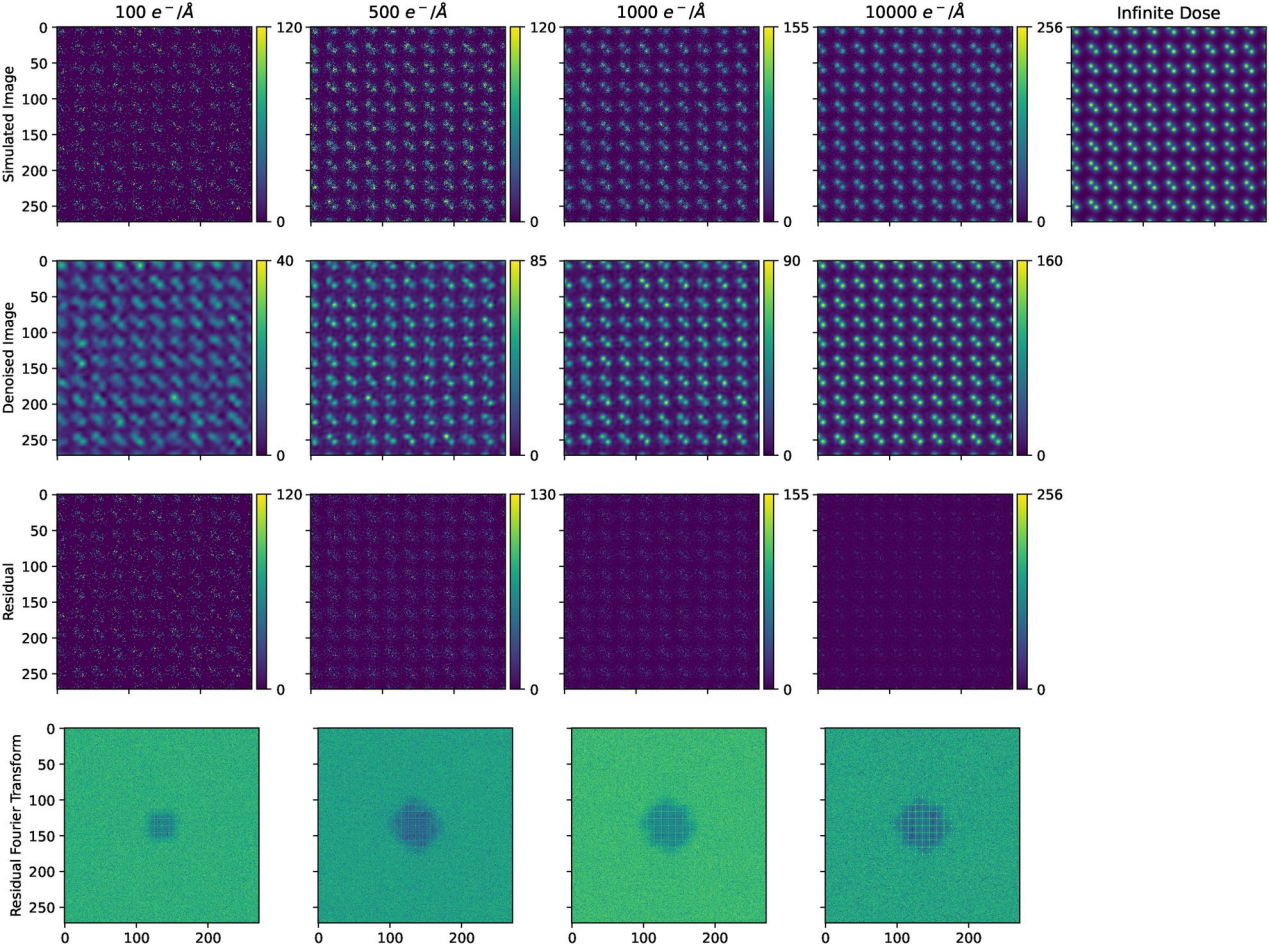
Reconstructions of simulations constructed using the Prismatic software package^45–47^, of the same SI [110] structure with different dosages, with Gaussian noise added with a standard deviation of 5% of the maximum beam current. In the lower dose, predominantly Poisson noise dominated regime, the algorithm has a smoothing effect. The especially “hot” pixels resulting from Poisson distributed noise have been clipped to enable a colour-range that brightens the simulated images to allow the reader a fairer qualitative assessment. In this predominantly Poisson noise simulated regime, the algorithm has a smoothing effect, and so the results are best presented with a different colour range.

The algorithm demonstrates robust performance across a wide range of dose-rates (100–10,000 e/Å$^{2}$), as illustrated in Fig. 5, with particular effectiveness for electron microscopy operators at dose-rates as low as 500 e/Å$^{2}$. Traditional quantitative metrics including Peak Signal-to-Noise Ratio (PSNR), Structural Similarity Index (SSIM), and Image Enhancement Factor (IEF) were calculated for comprehensive assessment, as shown in Table 1. PSNR measures the peak signal-to-noise ratio between original and processed images in decibels (higher is better), SSIM assesses structural similarity by comparing luminance, contrast, and structure patterns (ranging from 0 to 1, with 1 being identical), and IEF quantifies enhancement by measuring the ratio of noise reduction to original noise level (values above 1 indicate improvement).

**Table 1:** Conventional measures of denoising performance, such a Peak Signal to Noise Ratio (PSNR), Structural Similarity Index (SSIM), and Image Enhancement Factor (IEF) evaluated on the SI [110] structure at the different dose-rates pictured in Fig. 5.

e/Å	PSNR	SSIM	IEF
100	23.23 dB	0.2721	1.102
500	23.43 dB	0.4124	1.206
1000	25.07 dB	0.5565	1.121
10000	26.09 dB	0.7043	1.210

Notably, our analysis revealed a significant discrepancy between these conventional metrics and human perception of image quality, as determined through our survey of 89 electron microscopy experts and 135 non-experts. This inconsistency between quantitative metrics and expert assessment was a key motivation for introducing the deep learning component, the flexibility of which could capture the complex subjective factors that define optimal image reconstruction. For instance, while the PSNR improved only modestly from 23.23 dB at 100 e/Å$^{2}$ to 26.09 dB at 10,000 e/Å$^{2}$, our expert survey indicated a substantial perceptual improvement in image quality across these dose rates. This finding underscores the limitations of conventional metrics for low-dose imaging applications and highlights the advantage of our perception-aligned approach that consistently preserves critical structural features while effectively removing noise, as confirmed by both visual inspection and expert evaluation.

**Fig. 6:**
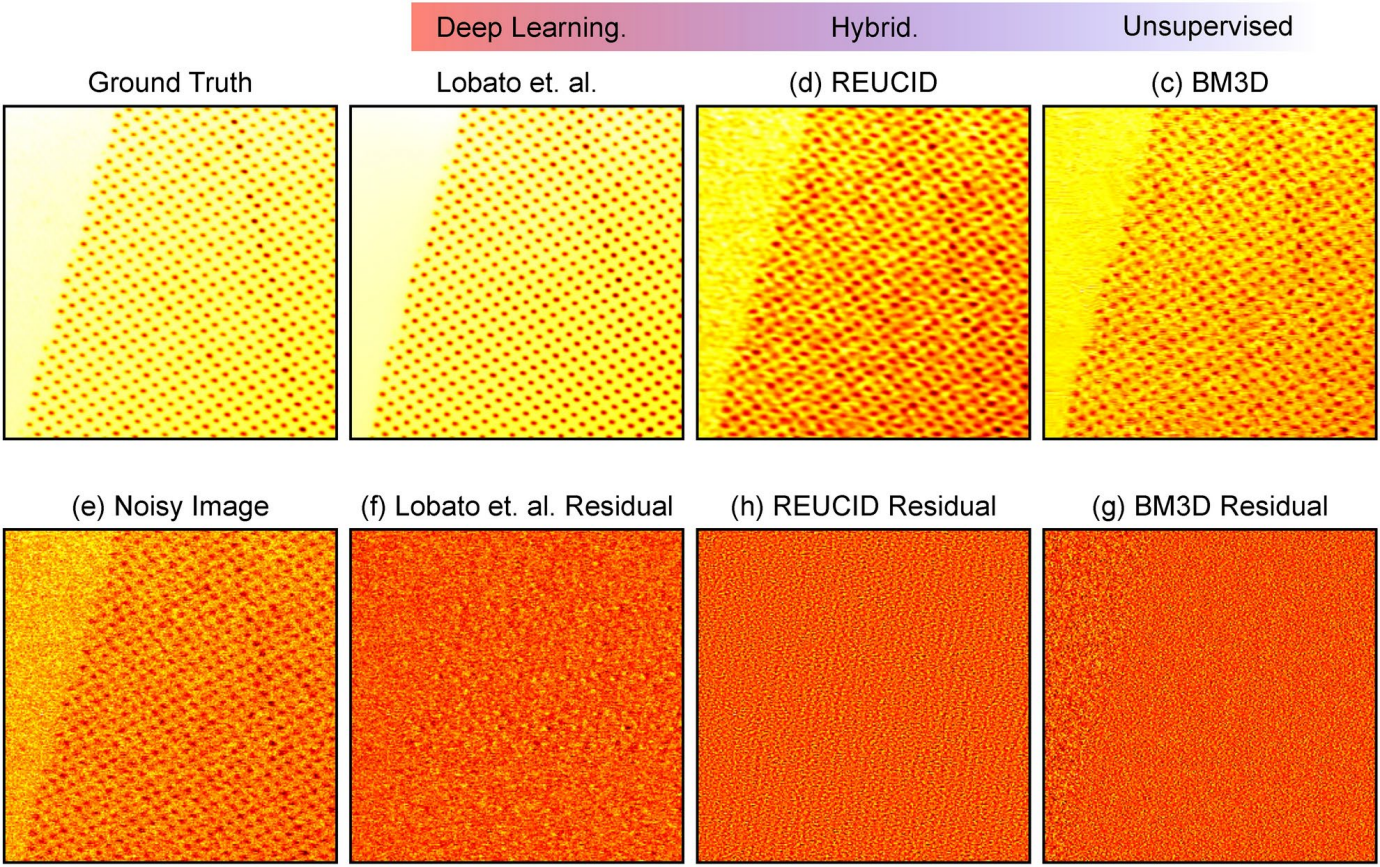
A comparison of REUCID with the current state-of-the-art, deep learning electron microscopy denoising algorithm by Lobato et al.^19^, and the classic BM3D^48^ denoising algorithm. The benchmark image is taken from the dataset of simulated images from the Lobato et al. paper. The residual image for a given algorithm is the difference between the noisy image and the algorithm’s reconstruction of the noisy image.

When comparing across denoising algorithms as in Fig. 6, we have presented the residuals between the reconstructions and the *noisy image*, rather than the ground truth which we have access to only when dealing with simulated data. The reason is that in the interest of maintaining the integrity of the experimental data, we are concerned with the removal of noise alone. If the information about the underlying structure is scarcely present in the noisy image, then our approach cannot reproduce it, because there is no deep learning component to infer from its training what should be present. This can be a positive or negative, depending on what is required. Models such as those by Lobato et al.^19^ do a very remarkable job of reproducing the ground truth, though the presence of structure in the residual tells us that they are not only removing nosie but actively inferring features based on learned priors. The REUCID architecture does not risk supressing genuine anomalies that do not conform to the training distribution, at the cost of potentially higher noise levels in regions where the signal-to-noise is extremely poor.

**Fig. 7:**
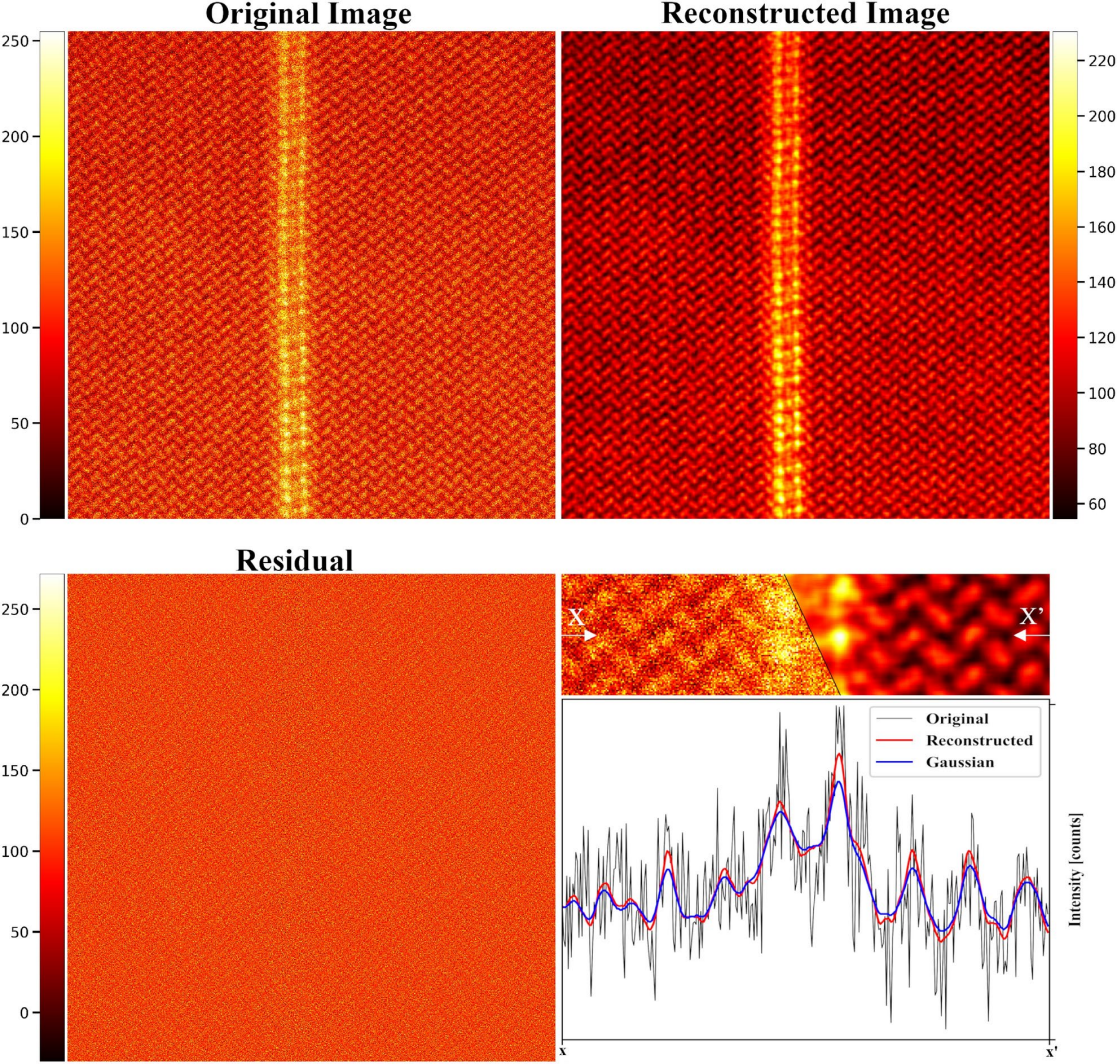
Reconstruction (top-right) of chiral Copper heterostructures templated by black Phosphorus (top-left), taken from previously published data shown in Ref.^49^. In the bottom-left, the difference between the original image and the reconstruction is plotted. In the bottom-right, a line plot along the direction marked in the image shows the superior peak structure of REUCID reconstructions compared to Gaussian smoothing.

The REUCID algorithm also demonstrates impressive performance on experimental images, see Figs. 1 and 7, with a plug-and-play approach, requiring no input from the user. Border artefacts or restrictions are not an issue due to the dense sampling towards the edges. At the lowest dose-rates tested (100 electrons per Angstrom squared), the “hot” Poisson-noise pixels are poorly represented in RSVD linear sum of average features; the net effect is these pixels are blurred, which appear as artefacts in the vacuum between atoms. It does, however, still offer a qualitative advantage over the untreated image.

### Computational efficiency

When running on a 11th Gen Intel(R) Core(TM) i7-11700, single threaded, a 256px $\times$ 256px image, such as those in Fig.5, takes 1.8 seconds. The noise level has no effect on denoising time. An 890px $\times$ 890px image takes 32 seconds on the same hardware for a full denoising on 5 patch stacks, though interim results could be returned using a lesser number of patch stacks to save time.

## Discussion

### Design philosophy

The design decisions which render the algorithm lightweight are exactly that which also ensures data integrity; focusing on image patches lets us discretise the workload to fit inside the computer RAM, while removing the possibility for global material structure to bias the denoising, improving generalisation to other materials. Exploiting the low-rank nature of microscopy images via the SVD step lets us simultaneously condense the denoising step to a computationally smaller task, and lets us restrict the ML component to a purely classification role, preventing it from over-imposing. Adaptive determination of the PSF size lets us standardise the patch features scale presented to the ML component, enabling a smaller model with better performance across materials; in direct contrast to the fixed kernel sizes used in conventional CNNs regardless of the PSF size in the underlying image. In trying to avoid having to feature engineer on the fly to facilitate eigenpatch clustering, we have displaced the eigenpatch utility determination to the imaging experts who informed the CNN training set outside of the live execution. The training protocol for the CNN is also built to work in the absence of simulated data and *a priori* knowledge of the noise profiles, in contrast to the deep learning approaches.

### Informing a qualitatively-aligned metric

Since this is a software aimed at assisting the technician during acquisition, the design decision was made to base the classification around human input. However, relying solely on human-labelled data would present a serious bottleneck in the algorithm’s further improvement in later work. With this in mind, we looked towards devising an edition of an existing denoising metric so that it might closely match the average expert response. With this new metric, the enlarging of the training set can be automated. Four different metrics were investigated for their suitability for editing; Peak Signal to Noise Ratio (PSNR), Mean Square Error (MSE), Structural Similarity Index (SSIM), and the Image Enhancement Factor (IEF).

For a given image used in the survey, the average response was taken to be the ideal number of eigenpatches to use in the reconstruction (this is the information the current model is built using). With this in mind, the same image was serially denoised, generating a set of denoised images where between 1 and 25 eigenpatches were used in the reconstruction. One of the 4 aforementioned denoising metrics was applied to the set of denoised images. Each metric would point to particular reconstruction out of the set of 25 as being the most optimally denoised; this candidate reconstruction would more often than not disagree with what the survey deemed to be the optimal reconstruction. Of the 4 metrics investigated, the IEF was chosen as the metric to edit, since it was most consistently closer to the survey average, and it could best discern a reconstruction using $\alpha$ eigenpatches from those which used $\alpha -1$ or $\alpha +1$.

In order to have the the optimal reconstruction deemed by the IEF match the survey response, an edition was made. The IEF was divided by a factor of $c^\alpha$, where $\alpha$ is the number of eigenpatches, and *c* was determined by a least squares analysis to be 0.64. With this edited denoising metric, the training set may be enlarged without having to rely on survey input any further.

### Breadth of scope

In the landscape of imaging modalities this may apply to, the patchisation step involved necessitates some amount of self-similarity in the images being denoising to be effective. The special case of HAADF electron microscopy images are particularly self-similar, a feature which is exploited directly in the PSF-estimation step. This step could easily be swapped out for an alternative method for kernel-size determination.

The most pressing generalisation bottleneck is in the CNN component. The efficacy of the classifier lies in what it has already been exposed to in its training, so porting over to a new imaging modality would necessitate a broader dataset. In terms of algorithm structure, having the CNN act as a classifier of small images significantly cuts down the size of the network, and hence the size of the dataset required to train the CNN. Additionally, the small data requirement is poised to make the most efficient use of human-annotated data, which is key since this algorithm is structured around data-fidelity in a live experimental setting. It is also worth noting that the dataset for training a CNN for this task can be developed in the absence of simulated images and noise profiles.

### Improving computational efficiency

In future work we aim to report on its integration into an experimental setup, and to push the algorithm speed towards real-time denoising to aid in experimental acquisition. To achieve real-time performance, the algorithm must be rewritten to exploit the potential for GPU acceleration of the SVD step^50,51^, and other available parallelisations of for loops and patch extractions/placements^52^.

## Conclusion

In this article, we have introduced a new, lightweight denoising architecture with the aim of aiding with data acquisition in low-dose, low-rank imaging regimes. By incorporating the SVD eigenvector discrimination step into a quickly-predicting neural network, the unavoidable computational workload associated with determining the useful eigenvectors has been delegated to the ML learning phase, outside of live execution. The algorithm is also structured such that the ML plays only a classification role; generative-type ML risks the introduction of artefacts from the training set. In this sense, the ML does not interact with the data, a crucial feature from an experimentalist’s perspective. Adaptive patch-size determination standardises the feature scale presenting to the classifier, reducing the complexity and training set required of the classifier. The patchisation and dense random sampling facilitates workload division such that the algorithm may be executed entirely within the RAM, and returning interim results, where a fast “rolling” overlap calculation quickly recontextualises the data in terms of patch overlap from different workload divisions.

Given the intended eventual experimental deployment, the training data for the survey was derived from expert consensus from around the globe, rather than the results of a quantitative metric; though an edited version of the Image Enhancement Factor has been identified as a suitable means to enlarge the training set moving forward. This will be necessary to expand the generality of the algorithm. We show that the architecture demonstrates impressive denoising and inpainting capacities for very low dose electron microscopy. When the noise is removed, the retrievable information is presented more clearly and enhanced, leading to a perceptible improvement, enabling the user to view further into what is resolvable.

## Data Availability

The dataset underlying the REUCID algorithm is publicly available on Zenodo with the following Digital Object Identifier (DOI): 10.5281/zenodo.13930575. The algorithm code is available on request from the lead author, Michael Mitchell, who can be contacted via email at mitchemi@tcd.ie.
